# Ecuadorian Cancer Patients’ Preference for Information and Communication Technologies: Cross-Sectional Study

**DOI:** 10.2196/jmir.8485

**Published:** 2018-02-20

**Authors:** Ivan Cherrez Ojeda, Emanuel Vanegas, Michell Torres, Juan Carlos Calderón, Erick Calero, Annia Cherrez, Miguel Felix, Valeria Mata, Sofia Cherrez, Daniel Simancas

**Affiliations:** ^1^ Universidad Especialidades Espíritu Santo Samborondón Ecuador; ^2^ Respiralab Research Group Guayaquil Ecuador; ^3^ Centro de Investigación en Salud Pública y Epidemiología Clínica Facultad de Ciencias de la Salud Eugenio Espejo Universidad Tecnológica Equinoccial Quito Ecuador

**Keywords:** social media, telemedicine, cancer, Web 2.0, mHealth

## Abstract

**Background:**

The instantaneous spread of information, low costs, and broad availability of information and communication technologies (ICTs) make them an attractive platform for managing care, patient communication, and medical interventions in cancer treatment. There is little information available in Latin America about the level of usage of ICTs for and by cancer patients. Our study attempts to fill this gap.

**Objective:**

The aim of this study was to assess the level of ICT use and patterns of preferences among cancer patients.

**Methods:**

We conducted an anonymous cross-sectional survey study in 500 Ecuadorian cancer patients. This questionnaire consisted of 22 items about demographic and clinical data, together with the preferences of people who use ICTs. Chi-square, crude, and adjusted logistic regressions were performed.

**Results:**

Of the total, 43.2% (216/500) of participants reported that they had access to the Internet, and 25.4% (127/500) reported that they neither owned a cell phone nor did they have access to the Internet. The Internet constituted the highest usage rate as a source of information about malignant diseases (74.3%, 162/218) regardless of age (*P*<.001). With regard to the preferences on how patients would like to use ICTs to receive information about diseases, WhatsApp (66.5%, 145/218) and short message service (SMS) text messaging (61.0%, 133/218) were widely reported as interesting communication channels. Similarly, WhatsApp (72.0%, 157/218) followed by SMS (63.8%, 139/218) were reported as the preferred ICTs through which patients would like to ask physicians about diseases. Adjusted regression analysis showed that patients aged between 40 and 64 years were more likely to be interested in receiving information through SMS (odds ratio, OR 5.09, 95% CI 1.92-13.32), as well as for asking questions to physicians through this same media (OR 9.78, CI 3.45-27.67) than the oldest group.

**Conclusions:**

WhatsApp, SMS, and email are effective and widely used ICTs that can promote communication between cancer patients and physicians. According to age range, new ICTs such as Facebook are still emerging. Future studies should investigate how to develop and promote ICT-based resources more effectively to engage the outcomes of cancer patients. The widespread use of ICTs narrows the gap between cancer patients with restricted socioeconomic conditions and those with wealth and easily available technological means, thereby opening up new possibilities in low-income countries.

## Introduction

### Background

A rapid increase in the use of information and communication technologies (ICTs) in recent decades is an enormous contributing factor in the development of a number of novel clinical and public health intervention strategies at every level, such as primary prevention, screening, early diagnosis, treatment, survivorship, and end-of-life care [1,2].

ICTs are broadly defined as technologies used to communicate, manipulate, and store data by electronic means [3]. These include email, short message service (SMS) text messaging, video chat, Web-based social media, as well as all the different computing devices that perform a wide range of communication and information functions [3].

ICTs might be beneficial for cancer patients in several ways. Web-based communities with low survival rates can provide emotional support, whereas those with high survival rates offer a more informational support [4]. For those looking for social support, blogs have proven to be helpful, especially on patients who become isolated because of physical deterioration or treatment requirements [5]. As a matter of fact, disclosing negative emotions or insights has proved to provide health benefits by reducing cancer concerns at follow-up, as reported in breast cancer patients [6]. Similar results could be expected to be found in other malignancies. On the other hand, for those looking for a more informational support, communities might offer advice on what to expect and what to do, or offer recommendations in common problems addressing test results, term definitions, treatment, time courses, side effects, and more [7].

There has been an increase in the number of publications about public health uses of social networking sites in the past 5 years [8]. This fair amount of studies regarding the impact and utility of ICTs emphasizes the importance and potential uses that the ICTs provide to the patient-physician relationship.

### Objectives

The objectives of this study were to assess the frequency of use of ICTs and to examine patterns of preferences among patients with any kind of cancer diagnosis. This study analyzes in cancer patients whether there is an existing association between ICTs’ use frequency and interest in receiving information and asking physicians about the disease through them. To our knowledge, there are no data about the use of ICTs in cancer patients for health-related purposes in Ecuador or Latin American countries. Understanding the role of ICTs in the context of a cancer patient could assist in the development of new personalized apps, promote the relationship between the patient and health care providers, and if used correctly, perhaps improve outcomes in the management of cancer treatment.

## Methods

### Study Design

We conducted an anonymous cross-sectional survey study in which 500 cancer patients rated themselves using questions about their level of ICT usage. Eligible outpatients from either public or private practices in SOLCA Hospital, a cancer center of reference in Ecuador, were surveyed using a Spanish version of the Michigan questionnaire (MQ), which was modified to be used by cancer patients. The survey included 22 items and collected information about demographics, use of cell phones, the interest of patients in using ICTs to receive information about cancer, and the interest of patients in using ICTs to communicate with health care providers about cancer.

Eligibility was restricted to patients aged 18 years and older who had been diagnosed with cancer. Patients younger than 18 years were not included because of a hospital policy restricting participation of minors, unless the parent or legal guardian signs a consent, and it is further revised and approved by a hospital official. We excluded patients with psychiatric diseases, language impairment, or those who found it difficult to visualize the survey.

### Sample Size

We used the Web-based Open Source Epidemiologic Statistics for Public Health (OpenEpi) to calculate the sample size for a descriptive study. Setting a population size of 1 million, an anticipated frequency of 50%, confidence interval of 95% and a design effect of 1.0, the calculated sample size was 384. We included 500 patients to increase power and overcome type-II error in anticipation to missing data.

### Procedure

We used a modified version of MQ, a self-administered survey that was hand-filled by the patient [9]. MQ was originally designed to determine the use of electronic media in asthma patients. We adopted a rigorous method to translate it into Spanish [10]. An expert panel of oncologists checked the adapted version. They also considered additional potential questionnaire items to include all necessary questions (Textbox 1). This questionnaire assesses the frequency of use of ICTs by patients and their preferences for receiving disease-related information.

We collected demographic information about each patient, including age, gender, education, and race/ethnicity, as well as information about duration of disease since diagnosis and the use of cancer medication. Furthermore, patients were asked whether they had access to the Internet and cell phone. Patients were also asked whether they had a smartphone.

Participants were then asked to quantify their use of each technology (SMS text messaging, Facebook, Twitter, email, LinkedIn, YouTube, Skype, WhatsApp, and the Internet) using a scale of daily, at least once a week, at least once a month, less than once a month, or never. They were also asked which ICTs (Internet, Facebook, Twitter, YouTube, email, and WhatsApp) they use to obtain information by themselves about cancer. Of note, the term Internet represents the networking communications systems used by the patient for the health-related purposes we analyze. The use of the Internet might be from any type of hardware that uses any Web-based platform, excluding Facebook, Twitter, YouTube, email, and WhatsApp; for instance, a Web browser, any social media not included in the categories, etc.

**Modifications and added questions suggested for the questionnaire.**

Modifications

- Question #6 of the Michigan Questionnaire addressing asthma treatment has been replaced by another addressing cancer treatment.
- Question #7 of the Michigan Questionnaire addressing ICT use frequency has been modified to include YouTube, LinkedIn, Skype, WhatsApp, and Instagram. On the other hand, MySpace was removed as a category.
- Question #9 of the Michigan Questionnaire addressing ICT use to get information about asthma has been modified to include YouTube, WhatsApp, Instagram, and others. MySpace was removed as a category.
- Questions #10 and #11 of the Michigan Questionnaire addressing interest in using ICTs to receive information and ask doctors about asthma, respectively, have been modified to include LinkedIn, WhatsApp, and others. MySpace was removed as a category.
- The disease of interest of the Michigan Questionnaire (asthma) has been replaced by cancer on questions 6, 9, 10, 11, 12, and 13.

Added Questions

- Included questions addressing patient’s occupation, education level, primary cancer location, present/absent metastasis, cellular and smartphone ownership, and access to Internet.

Participants were also asked to quantify their interest in receiving information about factors that could affect cancer control through ICTs. The level of interest was quantified as high, somewhat, low, or no interest. Using the same scale, participants were asked to quantify their interest in asking questions to their doctors or other health care providers using each of the ICT forms (SMS text messaging, Facebook, Twitter, LinkedIn, email, and WhatsApp). Free-text entries were solicited to determine what information participants would like to receive the most via these technologies, the reasons they might not be interested in using such technologies for communication, and any other comments about the use of these methods for cancer patients.

We incorporated WhatsApp as a new category into some questions in the survey. This category was included because we considered it highly relevant because of its penetration in Latin America, which according to the Global Web Index reaches 66% of the population, being the highest proportion reported among all continents [11]. This ICT is not included in the original questionnaire.

Before answering our questionnaire, patients were informed of the purpose of the study and their role in it. During the survey, patients completed their questionnaires either by themselves or with the help of a previously trained person (eg, physician, nurse, or intern). In total, we administered 673 surveys to cancer patients. However, 153 patients did not want to participate and left the survey in blank. After reaching 500 completed surveys, no more surveys were delivered. The response rate was 74.29%.

### Ethical Considerations

This study was approved by the Ethics Committee of the Hospital Luis Vernaza, Ecuador. We obtained informed consent before participation in the survey. We guaranteed that the identity of the patient would not be revealed.

### Statistical Analysis

For each ICT type, the frequency-of-use responses were dichotomized into categories of *at least once a week* and *less than once a week*. Age, gender, education level, years since the cancer diagnosis, and metastasis were used as independent variables on each analysis. Age groups were categorized into young adults (18-39 years), old adults (40-64 years), and elderly (≥65 years). Gender was either male or female. Education level was categorized into none or primary school, secondary school, and undergraduate or postgraduate. The time since cancer diagnosis was categorized as <3 years and ≥3 years. Metastasis was dichotomized into *yes* and *no*.

We performed a chi-square test to assess the association among Internet access or owning a cell phone or smartphone and age, gender, education level, and years since diagnosis. We employed the same test to determine the association between the same independent variables and the frequency of use of each ICT type (SMS text messaging, Facebook, Twitter, YouTube, email, the Internet, LinkedIn, Skype, and WhatsApp). We used the same analysis to determine whether there was an association between these independent variables and the use of each ICT to obtain information.

We performed similar analyses on the association between the independent variables described and the degree of interest (dichotomized into *high or some interest* and *little or no interest*) in receiving information through each ICT type and having high or some interest in communicating (asking physician) through each media type.

We undertook adjusted regression analyses between the complete set of independent variables and use (*at least once a week* and *less than once a week*) and interest in receiving information and communicating through each ICT. Analysis was adjusted for age, gender, education level, years since diagnosis, and metastasis. Reference categories were as follows: ≥65 years, male, no education/primary school, ≥3 years since diagnosis, and *yes* regarding the positive diagnosis for metastasis. Finally, we performed separated nonadjusted analysis between frequency of use of an ICT and interest in receiving information and communicating with physician through that same ICT.

All data were analyzed using the SPSS, version 24.0 software (SPSS Inc., Chicago, IL, USA). We performed Fisher exact test where necessary. A *P* value of less than .05 was considered statistically significant.

## Results

**Figure 1:**
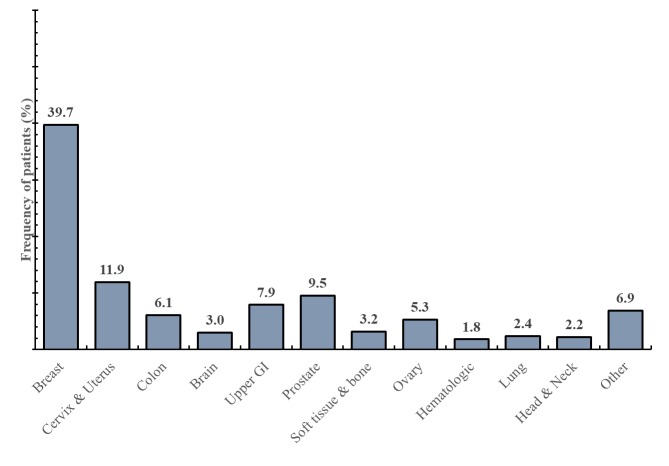
Types of malignancies in surveyed population.

From 500 patients, 389 (77.8%, 389/500) were female, and 170 (34.0%, 170/500) had a high school degree (Table 1). The average age was 57.5 years (standard deviation, SD 14.9), with an average time of being diagnosed with cancer of 2.9 years (Table 2). The most common type of malignancy was breast cancer, which was found in 196 patients (39.2%, 196/500; Figure 1).

### Internet Access, Owning a Cell Phone or Smartphone

Of the total number of participants, 216 (43.2%, 216/500) reported having access to the Internet. A total of 371 participants reported they owned a mobile phone (74.2%, 371/500), of which 42.9% (159/371) were smartphones (Table 3). Also, 127 participants reported of neither owning a cell phone nor having access to the Internet (25.4%, 127/500).

### Use of ICTs At Least Once a Week

Interestingly, WhatsApp presented the highest rate of ICT usage at least once a week (76.2%, 166/218), followed by Facebook (67.4%, 147/218), SMS text messaging (63.8%, 139/218), and the Internet (60.1%, 131/218; Table 3). SMS text messaging, Facebook, and Instagram were the most used ICTs by patients aged under 40 years (*P=*.002; Table 3). WhatsApp presented the highest rate of usage at every educational level (*P*=.01; Multimedia Appendix 1). Patients who had cancer for 3 years or more reported higher usage rates of WhatsApp (95%, 37/39), email (51%, 20/39), and Twitter (23%, 9/39; *P*=.05) than patients with fewer than 3 years of the disease (Multimedia Appendix 2).

### Use of ICTs for Seeking Cancer Information

**Figure 2:**
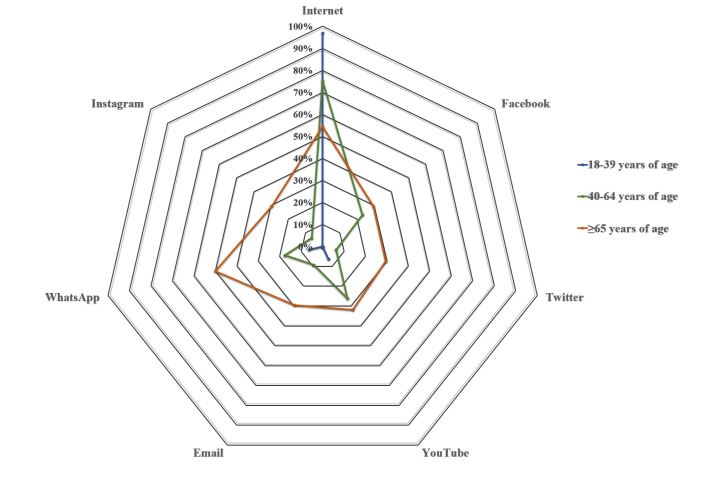
Preferences in using information and communication technologies to obtain information about disease by age groups.

Internet presented the highest usage rate as a source of information about cancer (74.3%, 162/218; Table 3, Figure 2). Other sources were YouTube (24.3%, 53/218), WhatsApp (22.5%, 49/218), and Facebook (21.1%, 46/218). Internet was the most used ICT for all age groups (*P*<.001; Table 3). WhatsApp was the most often referred-to form of ICT at every educational level (*P*=.001; Multimedia Appendix 1). In general, patients with 3 years or more of the disease used every ICT to look for information to a greater extent than did patients with fewer than 3 years of the disease (*P*=.04) (Multimedia Appendix 2).

**Table 1:** Demographic information of surveyed population (N=500).

Characteristics		Patients, n (%)
**Age in years**		
	18-39	62 (12.4)
	40-64	258 (51.6)
	≥65	180 (36.0)
**Gender**		
	Male	111 (22.2)
	Female	389 (77.8)
**Education level**		
	No education/Primary school	205 (41.0)
	Secondary school	170 (34.0)
	Undergraduate/Postgraduate	125 (25.0)
**Years with cancer**		
	<3	386 (77.8)
	≥3	110 (22.2)

**Table 2:** Mean age and years since diagnosis of surveyed population.

Characteristics		Mean (SD)
**Age in years**		57.5 (14.9)
	18-39	30.8 (6.8)
	40-64	53.3 (7.0)
	≥65	72.7 (5.7)
**Years with cancer**		2.9 (5.6)
	<3	0.9 (0.6)
	≥3	9.0 (9.0)

### High- or Medium-Level Interest in Receiving Disease Information Using ICTs

WhatsApp (66.5%, 145/218) and SMS text messaging (61.0%, 133/218) were widely reported as the most effective means of communication for receiving information, followed by Facebook (39.4%, 86/218), and email (38.1%, 83/218; Table 3). Across ages, WhatsApp was reported by most patients aged under 40 years (97%, 31/32) and older than 65 years (75%, 33/44), whereas SMS text messaging presented the highest interest rate in patients who were aged between 40 and 64 years (69.7%, 99/142; *P*<.001; Table 3). Meanwhile, all education levels were mostly interested in receiving information by WhatsApp (*P*<.001), followed by Facebook for those with secondary school degree or lower (*P*=.03; Multimedia Appendix 1). Individuals in both categories of years with cancer preferred WhatsApp for receiving information (*P*=.03; Multimedia Appendix 2).

### High- or Medium-Level Interest in Asking Physician’s Information Using ICTs

WhatsApp presented the highest interest rate for asking physicians information about the disease (72.0%, 157/218), followed by SMS text messaging (63.8%, 139/218), email (39.9%, 87/218), and Facebook (33.5%, 73/218; Table 3). Analysis by age reveals that the youngest and oldest groups prefer WhatsApp (97% and 80%, respectively; 31/32 and 35/44, respectively), whereas the middle group has the greatest affinity for SMS text messaging (71.8%, 102/142; *P*<.001; Table 3). In addition, both genders presented the highest interest rate for SMS text messaging (78%, 40/51 males and 59.3% (99/167), females; *P*=.01; Multimedia Appendix 3). When we turn to the results by educational level, the three categories showed the highest interest for WhatsApp (Multimedia Appendix 1).

### Logistic Regression Analysis

Patients aged under 65 years were associated with a higher chance of being interested in receiving information about cancer through Facebook when compared with those aged 65 years and older (reference category). In contrast to the reference, the odds of being interested in receiving information by SMS text messaging was higher in patients aged between 40 and 64 years (odds ratio, OR 5.09; Table 4).

With respect to the odds of interest in asking physicians for information by ICTs, SMS text messaging was the highest in patients who were aged between 40 and 64 years (OR 9.78), while Facebook was the most rated for the youngest group (OR 16.11; Table 4).

On the other hand, analysis by gender reveals that females were associated with less chances of being highly interested in receiving information through SMS text messaging (OR 0.22), email (OR 0.26), and WhatsApp (OR 0.27) than males (reference category), as well as less likely to be interested in communicating with physicians through SMS text messaging (OR 0.16) than the reference (Table 4).

**Table 3:** Use of information and communication technology (ICT) types to obtain information, interest in receiving information, and interest in asking a physician through ICT types about a disease, by age. All data are presented as percentages. Differences in values between the three age groups are significant at .05 significance level (N=500).

Questionnaire parameter		Age group in years, n (%)			*P* value	Total, n (%)
		18-39, n=62	40-64, n=258	≥65, n=180		
Internet access		32 (51.6)	141 (54.7)	43 (23.9)	<.001	216 (43.2)
**Phone owned**						
	Cell phone	52 (83.9)	202 (78.9)	117 (65.0)	<.001	371 (74.2)
	Smartphone	31 (96.9)	93 (66.9)	35 (89.7)	<.001	159 (75.7)
**Use of ICT type (at least once a week)**						
	SMS text messaging	18 (56.2)	103 (72.5)	18 (40.9)	<.001	139 (63.8)
	Facebook	29 (90.6)	95 (66.9)	23 (52.3)	.002	147 (67.4)
	Twitter	6(18.8)	9 (6.3)	4 (9.1)	.08^a^	19 (8.7)
	YouTube	14 (43.8)	48 (33.8)	13 (29.6)	.42	75 (34.4)
	Email	11 (34.4)	62 (43.7)	11 (25.0)	.07	84 (38.5)
	Internet	21 (65.6)	82 (57.8)	28 (63.6)	.61	131 (60.1)
	LinkedIn	0 (0.00)	6 (4.23)	0 (0.0)	.37^a^	6 (2.8)
	Skype	0 (0.00)	7 (4.93)	0 (0.0)	.20^a^	7 (3.2)
	WhatsApp	28 (87.5)	103 (72.54)	35 (79.6)	.16	166 (76.2)
	Instagram	13 (40.6)	13 (9.2)	9 (20.5)	<.001	35 (16.1)
**Uses of ICTs to obtain information about a disease**						
	Internet	31 (96.9)	107 (75.4)	24 (54.5)	<.001	162 (74.3)
	Facebook	0 (0.0)	33 (23.2)	13 (29.5)	.004	46 (21.1)
	Twitter	0 (0.0)	9 (6.3)	13 (29.5)	<.001^a^	22 (10.1)
	YouTube	2 (6.3)	37 (26.1)	14 (31.8)	.02	53 (24.3)
	Email	0 (0.0)	13 (9.2)	13 (29.5)	<.001	26 (11.9)
	WhatsApp	2 (6.3)	25 (17.6)	22 (50.0)	<.001	49 (22.5)
	Instagram	0 (0.0)	9 (6.3)	13 (29.5)	<.001^a^	22 (10.1)
**Interest in receiving information through ICT type (high/some interest)**						
	SMS text messaging	18 (56.3)	99 (69.7)	16 (36.4)	<.001	133 (61.0)
	Facebook	20 (62.5)	57 (40.1)	9 (20.5)	<.001	96 (39.4)
	Twitter	9 (18.8)	6 (6.3)	9 (13.6)	.05^a^	21(9.6)
	LinkedIn	3 (9.4)	9 (6.3)	4 (9.1)	.58^a^	16 (7.3)
	Email	11 (34.4)	54 (38.0)	18 (40.9)	.84	83 (38.1)
	WhatsApp	31 (96.9)	81 (57.0)	33 (75.0)	<.001	145 (66.5)
**Interest in asking physician through ICT type (high/some interest)**						
	SMS text messaging	21 (65.6)	102 (71.8)	16 (36.4)	<.001	139 (63.8)
	Facebook	20 (62.5)	46 (32.4)	7 (15.9)	<.001	73 (33.5)
	Twitter	3 (9.4)	9 (6.3)	6 (13.6)	.25^a^	18 (8.3)
	LinkedIn	0 (0.0)	0 (0.0)	4 (9.1)	.002^a^	4 (1.8)
	Email	13 (40.6)	54 (38.0)	20 (45.5)	.67	87 (39.9)
	WhatsApp	31 (96.9)	91 (64.1)	35 (79.5)	<.001	157 (72.0)

^a^Fisher exact test performed.

Furthermore, regression analysis by education level demonstrates that the highest degree category is not only less likely to be interested in receiving information about the disease through WhatsApp (OR 0.20) than the lowest degree category (reference category) but also are less likely to be interested in communicating with physicians through SMS text messaging (OR 0.34) and WhatsApp (OR 0.18) than the reference.

Moreover, when considering years with disease for the analysis, individuals with cancer for less than 3 years are more likely to use WhatsApp (OR 3.24) for putting questions to physicians about their disease when compared with patients with 3 years or more with cancer (reference category; Table 4).

In addition, patients with no metastasis are more likely to be interested in asking physicians about their disease through email (OR 2.24) than those with metastasis (reference category). However, they were also less likely than the reference for other outcomes.

Finally, individuals using SMS text messaging, email, and WhatsApp at least once a week were associated with more chances of receiving and asking information through these media than individuals using them for less than once a week (reference category; Table 4).

**Table 4:** Characteristics of frequent users of selected electronic media type (≥1 time/week) showing high/some interest in receiving information and asking physicians about cancer. Regression analysis was adjusted for variables such as age, gender, education level, years with cancer and metastasis. Regression analysis using weekly information and communication technology (ICT) types was performed separately (nonadjusted).

Variable			Interest in receiving information through ICT type, OR (95% CI)	Interest in asking physicians through ICT type, OR (95% CI)
**SMS text messaging (n=218)**				
	**Age in years**^a^			
		18-39	1.81 (0.56-5.84)	3.37 (0.99-11.46)^b^
		40-64	5.09 (1.95-13.32)^b^	9.78 (3.45-27.67)^b^
	**Gender**^c^			
		Female	0.22 (0.09-0.52)^b^	0.16 (0.06-0.43)^b^
	**Education level**^d^			
		Undergraduate/Postgraduate	0.95 (0.39-2.34)	0.34 (0.13-0.93)^b^
	**Metastasis**^e^			
		No metastasis	0.39 (0.18-0.82)^b^	0.58 (0.27-1.23)
	Weekly SMS use^f,g^		10.27 (5.40-19.54)^b^	10.98 (5.73-21.05)^b^
**Facebook (n=218)**				
	**Age in years**			
		18-39	11.53 (3.13-42.46)^b^	16.11 (4.06-63.86)^b^
		40-64	4.27 (1.49-12.21)^b^	4.42 (1.43-13.65)^b^
	**Metastasis**			
		No metastasis	0.35 (0.17-0.72)^b^	0.44 (0.22-0.90)^b^
**Email (n=218)**				
	**Age in years**			
		40-64	0.53 (0.20-1.41)	0.29 (0.12-0.71)^b^
	**Gender**			
		Female	0.26 (0.12-0.55)^b^	0.74 (0.37-1.50)
	**Metastasis**			
		No metastasis	1.94 (0.96-3.92)	2.24 (1.13-4.42)^b^
	Weekly email use		3.17 (1.79-5.63)^b^	3.537 (1.993-6.29)^b^
**WhatsApp (n=218)**				
	**Age in years**			
		40-64	0.30 (0.09-1.05)	0.16 (0.03-0.82)^b^
	**Gender**			
		Female	0.27 (0.11-0.66)^b^	1.08 (0.46-2.57)
	**Education level**			
		Undergraduate/Postgraduate	0.20 (0.07-0.60)^b^	0.18 (0.05-0.64)^b^
	**Years with cancer**^h^			
		<3 years with cancer	0.28 (0.11-0.75)^b^	>3.24 (1.28-8.17)^b^
	Weekly SMS use		96.63 (27.87-335.078)^b^	12.69 (6.14-26.24)^b^

^a^Reference age category is ≥65 years.

^b^.05 significance level

^c^Reference gender category is male.

^d^Reference educational-level category is No education/Primary school.

^e^Reference metastasis category is *presence of metastasis*.

^f^SMS: short message service.

^g^Reference ICT use category is *less than once a week*.

^h^Reference *years with cancer* category is ≥3 years.

## Discussion

### Principal Findings

This study analyzed the preferences of ICTs among cancer patients when used for health-related purposes. Existing associations between ICTs use frequency and interest in receiving information and communicating with physicians has been highlighted.

For instance, WhatsApp was the best rated ICT for both purposes, followed by SMS text messaging. Females and high-education degree patients were less likely to be interested in using these ICTs for such purposes. Also, adult patients were more likely to be interested in using Facebook for the purposes described than the elderly. To add, the Internet (Web browsing) was the most used ICT to look for information about cancer, regardless of age.

### Internet Access, Cell Phone, and Smartphone Possession

Research indicates that oncologic patients tend to use the Internet to find information about their disease and receive social support [12]. It has been previously reported by Keinki et al that 59% of cancer patients use the Internet and 7.5% use it as a primary source of health information [13]. A recent publication determined that 43.4% of all Latin-American households were connected to the Internet in 2015 [14]. Our data fit the former distribution as 43.2% of our sample has access to Internet. However, there are populations with no or little access to the Internet as well as without or limited skills to use it effectively [15]. These issues limit their access to health information.

Certainly, the proliferation of ICT users with mobile devices provides an opportunity for easy access to medical information [16]. Recently, a publication found that improving health media strategy can be effective for achieving health equity in any given society [17]. One quarter of our participants reported neither owning a cell phone nor having access to Internet. This group misses out on the opportunity to have any kind of health intervention through ICTs.

Moreover, the use of smartphones has grown in Latin America [18]. Coughlin and coinvestigators showed the potential for research-tested smartphone apps to provide a low-cost and effective strategy for preventing breast cancer in women [1]. In our study, approximately one-third of patients have a smartphone. Thus, the possession of a smartphone is proven to be less attractive for applying interventions in low-income countries because of poor adoption for this technology.

### Receiving Information and Communicating With Physicians

Our results suggest that SMS text messaging for people aged 40 to 64 years is a useful tool for communicating (both for receiving and asking for information) with cancer patients. Patients aged 40 to 64 years have a 5 times greater chance of being interested in receiving information by SMS text messaging and almost a 10 times greater chance of asking physicians about their disease, and SMS text messaging is the most highly rated of all other media within this age group.

There are several practical reasons for using SMS text messaging. It costs less than voice messaging, and it can reach people whose phones are switched off. Furthermore, SMS text messaging is silent, which means that messages can be sent and received in places where it may be impractical to hold a conversation [19].

WhatsApp is a cross-platform instant messaging app that allows smartphone users to exchange text, image, video, and audio messages. In Latin America, around two-thirds of Internet users are *Whatsapping* compared with North Americans, who barely use the app [11].This platform was the most extensively used by our cancer patients. The same reasons that justify SMS text messaging usage theoretically apply for WhatsApp use. Furthermore, several Wi-Fi access points in different places throughout the city may facilitate WhatsApp usage. WhatsApp should be the main focus between all ICTs to facilitate medical advice and support.

Currently, in Latin America some mobile operators offer different plans that include unlimited WhatsApp and Facebook services. This is presumably the reason why Latin America has a higher Internet penetration rate when compared with other regions [20].

It is known that older adults are often portrayed as less avid users of ICTs. However, we found that older patients also use WhatsApp for receiving information and for asking their physician about cancer. This goes along with a recent publication in which WhatsApp was declared to be the most frequently used app with an average of 26.4 accesses per day. WhatsApp is a very relevant app, as it is always associated to a flat rate bill, has no limitations, and is cheaper than phone calls or SMS text messages [21].

The results of this study suggest that WhatsApp is a useful tool for communicating (both for receiving and asking for information) with cancer patients. WhatsApp showed the highest level of interest in receiving information and asking physicians across all categories of age, education level, and years with cancer. Therefore, the development of adequate text messages for SMS and WhatsApp to support and inform cancer patients is the next challenge. It is key to determine which is the most adequate messaging service to encourage the diffusion of health information.

In general, WhatsApp and SMS text messaging were reliable communication channels for all ages. In young people and patients with fewer than 3 years of cancer, Facebook was especially remarkable. It is known that cancer campaigns come equipped with several photos and have produced the most significant engagement rate, suggesting that visual content may be more effective in facilitating engagement in public health social and digital media campaigns [22]. The rapid diffusion, low costs, and broad availability of social media make it an attractive platform for managing care, communication, and interventions in cancer.

Moreover, this study showed that approximately 75% of patients use the Internet as a source of information about their disease. The Internet is a common venue for disseminating and accessing health information [23].

In addition, by attempting to reduce risks toward cancer and disseminating evidence-based information about cancerous diseases in combination with a minimal cost, YouTube has shown to be the ideal media form [24,25]. YouTube is used by 24.3% of our patients to obtain information about cancer. However, it must be remembered that not all videos in chronic diseases are uploaded by credible authoritative sources [26].

On the other hand, it has been reported that elderly people with cancer are not familiar with YouTube [27]. Older people have reported that they tend to restrict heavy media consumption when using their mobile data plan to control their budget, providing this as a reason not to use YouTube [21]. In contrast with these data, our study found that almost one-third of our patients older than 65 years used YouTube to obtain information about cancer, and when compared with the other age groups, it presented the highest rate of use out of all ICT forms. Thus, our results encourage the continued use of this kind of communication channel in this age group. In the future, it is necessary to understand why the elderly cancer patients have an interest in using YouTube to look for information about cancer.

### Limitations

Even though ICTs may provide several benefits for the patient and health communication, there are some limitations that might actually be deleterious for health care [28]. For example, the quality of the content and reliability of health information is sometimes questionable [29,30]. In some instances, authors are unknown [29]. Also, in the scenario of a patient-physician communication through email, confidential information and medical records might be vulnerable to security breaches [31]. Patients may accidentally share personal information through social media or provide incorrect advice, which can be harmful for other patients [32].

Our study has some limitations. First, it was not conducted in all Latin American countries and the preferred use of ICTs in other countries in the same region might differ. Second, almost 80% of the respondents were female; therefore, our findings are not simply extendable to males. In addition, approximately 40% of our patients were diagnosed with breast cancer and almost 12% with cervix and uterus malignancies; thus, the results cannot be extrapolated to other types of cancer. Moreover, the simple fact of applying a survey as the measure instrument may produce unreliable results because of variability on interpretation of the respondent, lack of awareness of what the survey is asking, the emotional status of the patient in the hospital setting, and missing data. Furthermore, our survey has not been validated. However, one strength of this study is that it covered a good sample size (N=500) of cancer patients. The sample also included participants of different age, sex, and educational level. To the best of our knowledge, our study is the first to explore the utility of WhatsApp in cancer patients, and our results provide evidence that this media is quite reliable. Also, ours is one of the first studies performed on the Latin American population concerning ICT usage in this disease. Future research is needed to confirm our findings and assess the real use of ICT tools.

Randomized trials will of course be necessary to determine the efficacy and cost-effectiveness of all new ICT tools in promoting cancer control for patients and support among these individuals, as well as providing a source of information about their disease and encouraging self-management. We also need to examine the benefit for cancer patients. However, the widespread use of ICTs opens up new possibilities for the relationship between physicians and patients.

### Conclusions

A variety of ICT forms are revolutionizing health care and becoming mainstream tools to assist patients in self-monitoring and decision making. In this study, we have determined that WhatsApp presented the highest rate of interest for receiving information and communicating with physicians, followed by SMS text messaging. The Internet represents the most significant source of information regardless of age, although patients need to be cautious as the content provided may be unreliable and deleterious for disease management. Depending on age, new ICTs such as Facebook and Twitter are still emerging. Recognizing patterns of preferences can be useful to target specific patient profiles better through ICTs. Future studies should investigate how to develop and promote ICT-based resources more effectively to engage the outcomes of cancer patients. The widespread use of ICTs opens new possibilities for cancer patients in developing countries. Furthermore, robust research is required to establish whether social media improves health communication practices in both short and long term.

## Appendix

Multimedia Appendix 1 Use, obtain information, interest receiving information, and interest in asking a physician through ICT types by education level.

Multimedia Appendix 2 Use, obtain information, interest receiving information, and interest in asking a physician through ICT types by years with cancer.

Multimedia Appendix 3 Use, obtain information, interest receiving information, and interest in asking a physician through ICT types by gender.

## Abbreviations

ICTs: information and communication technologies
MQ: Michigan questionnaire
SMS: short message service

## References

[1] Coughlin SS, Thind H, Liu B, Wilson LC (2016). Towards research-tested smartphone applications for preventing breast cancer. Mhealth.

[2] Panayi ND, Mars MM, Burd R (2013). The promise of digital (mobile) health in cancer prevention and treatment. Future Oncol.

[3] Perron BE, Taylor HO, Glass JE, Margerum-Leys J (2010). Information and communication technologies in social work. Adv Soc Work.

[4] Buis LR, Whitten P (2011). Comparison of social support content within online communities for high- and low-survival-rate cancers. Comput Inform Nurs.

[5] Kim B, Gillham DM (2013). The experience of young adult cancer patients described through online narratives. Cancer Nurs.

[6] Shim M, Cappella JN, Han JY (2011). How does insightful and emotional disclosure bring potential health benefits? Study based on online support groups for women with breast cancer. J Commun.

[7] Civan A, Pratt W (2007). Threading Together Patient Expertise. AMIA Annual Symposium Proceedings.

[8] Capurro D, Cole K, Echavarría M, Joe J, Neogi T, Turner A (2014). The use of social networking sites for public health practice and research: a systematic review. J Med Internet Res.

[9] Baptist AP, Thompson M, Grossman KS, Mohammed L, Sy A, Sanders GM (2011). Social media, text messaging, and email-preferences of asthma patients between 12 and 40 years old. J Asthma.

[10] Sperber AD (2004). Translation and validation of study instruments for cross-cultural research. Gastroenterology.

[11] Young K (2016). globalwebindex.

[12] Yli-Uotila T, Rantanen A, Suominen T (2013). Motives of cancer patients for using the Internet to seek social support. Eur J Cancer Care (Engl).

[13] Keinki C, Seilacher E, Ebel M, Ruetters D, Kessler I, Stellamanns J, Rudolph I, Huebner J (2016). Information needs of cancer patients and perception of impact of the disease, of self-efficacy, and locus of control. J Cancer Educ.

[14] Rojas E, Poveda L, Grimblatt N (2016). Digital Repository: Economic Commission for Latin America and the Caribbean.

[15] Rini C, Lawsin C, Austin J, DuHamel K, Markarian Y, Burkhalter J, Labay L, Redd WH (2007). Peer mentoring and survivors' stories for cancer patients: positive effects and some cautionary notes. J Clin Oncol.

[16] Jawad M, Abass J, Hariri A, Akl EA (2015). Social media use for public health campaigning in a low resource setting: the case of waterpipe tobacco smoking. BioMed Res Int.

[17] Ishikawa Y, Kondo N, Kawachi I, Viswanath K (2016). Are socioeconomic disparities in health behavior mediated by differential media use? Test of the communication inequality theory. Patient Educ Couns.

[18] Smith A (2015). Pewinternet.

[19] Kaplan WA (2006). Can the ubiquitous power of mobile phones be used to improve health outcomes in developing countries? Globalization and health. Global Health.

[20] Wharton School of the University of Pennsylvania (2013). Knowledge @ Wharton.

[21] Rosales A, Fernández-Ardèvol M (2016). Beyond WhatsApp: older people and smartphones. Revista Română de Comunicare şi Relaţii Publice.

[22] Theiss SK, Burke RM, Cory JL, Fairley TL (2016). Getting beyond impressions: an evaluation of engagement with breast cancer-related Facebook content. MHealth.

[23] Park S, Oh HK, Park G, Suh B, Bae WK, Kim JW, Yoon H, Kim DW, Kang SB (2016). The source and credibility of colorectal cancer information on Twitter. Medicine (Baltimore).

[24] Lauckner C, Whitten P (2016). The differential effects of social media sites for promoting cancer risk reduction. J Cancer Educ.

[25] Cooper CP, Gelb CA, Chu J (2016). Gynecologic cancer Information on YouTube: will women watch advertisements to learn more?. J Cancer Educ.

[26] Garg N, Venkatraman A, Pandey A, Kumar N (2015). YouTube as a source of information on dialysis: a content analysis. Nephrology (Carlton).

[27] Heo J, Chun M, Lee HW, Woo JH (2016). Social media use for cancer education at a community-based cancer center in South Korea. J Cancer Educ.

[28] Moorhead SA, Hazlett DE, Harrison L, Carroll JK, Irwin A, Hoving C (2013). A new dimension of health care: systematic review of the uses, benefits, and limitations of social media for health communication. J Med Internet Res.

[29] Adams SA (2010). Revisiting the online health information reliability debate in the wake of “web 2.0”: an inter-disciplinary literature and website review. Int J Med Inform.

[30] Kim S (2009). Content analysis of cancer blog posts. J Med Libr Assoc.

[31] Nordfeldt S, Hanberger L, Berterö C (2010). Patient and parent views on a Web 2.0 Diabetes Portal--the management tool, the generator, and the gatekeeper: qualitative study. J Med Internet Res.

[32] Adams SA (2010). Blog-based applications and health information: two case studies that illustrate important questions for Consumer Health Informatics (CHI) research. Int J Med Inform.

