# Supplementary material for: Ecuadorian Cancer Patients’ Preference for Information and Communication Technologies: Cross-Sectional Study
Source: J Med Internet Res. 2018 Feb 20;20(2):e50. doi: 10.2196/jmir.8485 (PMC5840480; doi:10.2196/jmir.8485)
Supplement: Multimedia Appendix 1 [file jmir_v20i2e50_app1.pdf]

Multimedia Appendix 1

Use, obtain information, interest receiving information, and interest in asking a physician through ICT types by education level.

	No education/ Primary (n= 205)	Secondary (n= 170)	Undergraduate/ Postgraduate (n= 125)	P value	Total (n= 500)
Internet access	24.9%	39.4%	78.4%	.000	43.2%
Owning					
Cellphone	59.5%	77.4%	95.2%	.000	74.5%
Smartphone	81.3%	84.1%	67.7%	.035	75.7%
Use of ICT type (at least once a week)					
SMS	68.6%	70.6%	56.6%	.128	63.8%
Facebook	62.7%	75.0%	64.6%	.268	67.4%
Twitter		4.4%	6.1%	.007	8.7%
YouTube	54.9%	5.9%	43.4%	.000	34.4%
Email	33.3%	27.9%	48.5%	.019	38.5%
Internet	60.8%	54.4%	63.6%	.486	60.1%
LinkedIn	11.8%	0.0%	0.0%	.000[a]	2.8%
Skype	0.0%	0.0%	7.1%	.019[a]	3.2%
WhatsApp	86.3%	82.4%	66.7%	.010	76.1%
Instagram	19.6%	17.6%	13.1%	.540	16.1%
Uses ICT to obtain information about disease					
Internet	68.6%	76.5%	75.8%	.566	74.3%
Facebook	31.4%	13.2%	21.2%	.056	21.1%
Twitter	21.6%	5.9%	7.1%	.008	10.1%
YouTube	33.3%	17.6%	24.2%	.142	24.3%
Email	21.6%	5.9%	11.1%	.031	11.9%
WhatsApp	41.2%	11.8%	20.2%	.001	22.5%
Instagram	21.6%	5.9%	7.1%	.008	10.1%
Interest in receiving information through ICT type (high/some interest)					
SMS	51.0%	67.6%	61.6%	.180	61.0%

Facebook	39.2%	51.5%	31.3%	.032	39.4%
Twitter	23.5%	4.4%	6.1%	.001	9.6%
LinkedIn	19.6%	4.4%	3.0%	.002[a]	7.3%
Email	35.3%	20.6%	51.5%	.000	38.1%
WhatsApp	82.4%	75.0%	52.5%	.000	66.5%
Interest in asking physician through ICT type (high/some interest)					
SMS	58.8%	75.0%	58.6%	.067	63.8%
Facebook	35.3%	32.4%	33.3%	.944	33.5%
Twitter	23.5%	0.0%	6.1%	.000	8.3%
LinkedIn	7.8%	0.0%	0.0%	.003[a]	1.8%
Email	39.2%	29.4%	47.5%	.064	39.9%
WhatsApp	86.3%	82.4%	57.6%	.000	72.0%

Notes: All data are presented as percentages. Differences in values between the three education level groups are significant at .05 significance level
