# Supplementary material for: Ecuadorian Cancer Patients’ Preference for Information and Communication Technologies: Cross-Sectional Study
Source: J Med Internet Res. 2018 Feb 20;20(2):e50. doi: 10.2196/jmir.8485 (PMC5840480; doi:10.2196/jmir.8485)
Supplement: Multimedia Appendix 2 [file jmir_v20i2e50_app2.pdf]

Multimedia Appendix 2

Use, obtain information, interest receiving information, and interest in asking a physician through ICT types by years with cancer.

	< 3 years (n= 386)	≥ 3 years (n= 110)	P value	Total (n= 496)
Internet	45.3%	34.5%	.044	42.9%
Owning				
Cellphone	77.6%	62.7%	.002	74.3%
Smartphone	74.3%	80.6%	.450	75.2%
Use of ICT type (at least once a week)				
SMS	65.7%	61.5%	.621	65.0%
Facebook	70.9%	59.0%	.148	68.7%
Twitter	5.7%	23.1%	.002[a]	8.9%
YouTube	36.6%	28.2%	.322	35.0%
Email	34.3%	51.3%	.047	37.4%
Internet	62.3%	46.2%	.064	59.3%
LinkedIn	3.4%	0.0%	.595[a]	2.8%
Skype	2.3%	7.7%	.116[a]	3.3%
WhatsApp	73.7%	94.9%	.004	77.6%
Instagram	16.6%	15.4%	.856	16.4%
Uses ICT to obtain information about disease				
Internet	77.7%	56.4%	.006	73.8%
Facebook	18.9%	23.1%	.548	19.6%
Twitter	5.1%	23.1%	.001[a]	8.4%
YouTube	21.1%	30.8%	.196	22.9%
Email	7.4%	23.1%	.007[a]	10.3%
WhatsApp	17.1%	38.5%	.003	21.0%
Instagram	5.1%	23.1%	.001[a]	8.4%
Interest in receiving information through ICT type (high/some interest)				
SMS	64.6%	51.3%	.122	62.1%
Facebook	43.4%	25.6%	.040	40.2%

Twitter	6.9%	23.1%	.005[a]	9.8%
LinkedIn	5.1%	17.9%	.013[a]	7.5%
Email	40.0%	23.1%	.048	36.9%
WhatsApp	64.6%	82.1%	.035	67.8%
Interest in asking physician through ICT type (high/some interest)				
SMS	68.6%	48.7%	.019	65.0%
Facebook	36.6%	23.1%	.108	34.1%
Twitter	5.1%	23.1%	.001[a]	8.4%
LinkedIn	0.0%	10.3%	.001[a]	1.9%
Email	40.6%	41.0%	.958	40.7%
WhatsApp	76.0%	61.5%	.065	73.4%

Notes: All data are presented as percentages. Differences in values between the two "years with cancer" groups are significant at .05 significance level
