# Supplementary material for: Ecuadorian Cancer Patients’ Preference for Information and Communication Technologies: Cross-Sectional Study
Source: J Med Internet Res. 2018 Feb 20;20(2):e50. doi: 10.2196/jmir.8485 (PMC5840480; doi:10.2196/jmir.8485)
Supplement: Multimedia Appendix 3 [file jmir_v20i2e50_app3.pdf]

Multimedia Appendix 3

Use, obtain information, interest receiving information, and interest in asking a physician through ICT types by gender.

	Male (n=111)	Females (n=389)	P value	Total (n=500)
Internet	45.9%	42.4%	.516[a]	43.2%
Owning				
Cellphone	64.9%	77.3%	.010[a]	74.5%
Smartphone	60.8%	80.5%	.008[a]	75.7%
Use of ICT type (at least once a week)				
SMS	66.7%	62.9%	.740[a]	63.8%
Facebook	52.9%	71.9%	.016[a]	67.4%
Twitter	3.9%	10.2%	.256[a]	8.7%
YouTube	35.3%	34.1%	.868[a]	34.4%
Email	51.0%	34.7%	.048[a]	38.5%
Internet	62.7%	59.3%	.745[a]	60.1%
LinkedIn	0.0%	3.6%	.340[a]	2.8%
Skype	0.0%	4.2%	.204[a]	3.2%
WhatsApp	74.5%	76.6%	.851[a]	76.1%
Instagram	19.6%	15.0%	.513[a]	16.1%
Uses ICT to obtain information about disease				
Internet	80.4%	72.5%	.279[a]	74.3%
Facebook	37.3%	16.2%	.003[a]	21.1%
Twitter	17.6%	7.8%	.060[a]	10.1%
YouTube	41.2%	19.2%	.003[a]	24.3%
Email	25.5%	7.8%	.002[a]	11.9%
WhatsApp	25.5%	21.6%	.568[a]	22.5%
Instagram	17.6%	7.8%	.060[a]	10.1%
Interest in receiving information through ICT type (high/some interest)				
SMS	74.5%	56.9%	.032[a]	61.0%
Facebook	43.1%	38.3%	.624[a]	39.4%

Twitter	3.9%	11.4%	.173[a]	9.6%
LinkedIn	0.0%	9.6%	.026[a]	7.3%
Email	66.7%	29.3%	.000[a]	38.1%
WhatsApp	74.5%	64.1%	.180[a]	66.5%
Interest in asking physician through ICT type (high/some interest)				
SMS	78.4%	59.3%	.013[a]	63.8%
Facebook	39.2%	31.7%	.397[a]	33.5%
Twitter	3.9%	9.6%	.255[a]	8.3%
LinkedIn	0.0%	2.4%	.575[a]	1.8%
Email	47.1%	37.7%	.255[a]	39.9%
WhatsApp	70.6%	72.5%	.859[a]	72.0%

Notes: All data are presented as percentages. Differences in values between the two gender groups are significant at .05 significance level
